# The impact of the COVID-19 pandemic response on other health research

**DOI:** 10.2471/BLT.20.257485

**Published:** 2020-07-06

**Authors:** Jerome Amir Singh, Sunita VS Bandewar, Elizabeth Anne Bukusi

**Affiliations:** a Centre for the AIDS Programme of Research in South Africa, University of KwaZulu-Natal, Private Bag X13, Congella, Durban 4013, South Africa.; b Health, Ethics and Law Institute of Forum for Medical Ethics Society, Mumbai-Pune, India.; c Center for Microbiology Research, Kenya Medical Research Institute, Nairobi, Kenya.

## Abstract

While governments have been focusing on the unprecedented disruption to the global economy caused by coronavirus disease 2019 (COVID-19) and the urgent need for COVID-19 research, other health research has become a casualty of the pandemic. Major research operations that are unrelated to COVID-19 have been significantly diminished or suspended entirely because of either COVID-19-related legal restrictions or logistical, staffing or operational concerns. Billions of people globally are currently affected by lockdowns or curfews. Since the timescale of such restrictive measures is unknown and subject to change, many studies are now in limbo and the welfare of tens of thousands of study participants is at risk. These circumstances have introduced complex ethical challenges that merit urgent attention from international sponsors, researchers and regulators. Certain sponsors and regulators have published guidelines on how the COVID-19-related disruptions to clinical research should be managed. Although these guidelines provide a good starting point in navigating the challenges of the evolving pandemic, they only apply to those researchers funded or governed by these bodies. Here, we provide guidelines on managing such disruptions that apply beyond these specific settings. We highlight some of the effects of the COVID-19 pandemic on other ongoing research projects that are unrelated to COVID-19 and provide practical guidance on how the welfare of affected study participants should be managed. We conclude that policy-makers, sponsors, researchers and regulators must adopt a more flexible approach to ensure participant safety, while maintaining data integrity and complying with good clinical practices.

## Résumé

Pendant que les gouvernements se concentrent sur les profonds bouleversements que la maladie à coronavirus 2019 (COVID-19) engendre pour l'économie mondiale ainsi que sur l'urgence des recherches qui y sont consacrées, d'autres études sont touchées de plein fouet par la pandémie. Plusieurs travaux de recherche d'importance majeure, sans lien avec la COVID-19, ont considérablement ralenti ou ont été totalement interrompus. Soit en raison des restrictions légales relatives à la COVID-19, soit à cause de problèmes logistiques, opérationnels ou d'effectifs. En ce moment, des milliards de personnes à travers le monde sont soumises à un confinement ou un couvre-feu. Comme la durée de telles mesures restrictives est inconnue et susceptible de changer, de nombreuses études sont actuellement en suspens et le bien-être de dizaines de milliers de participants est menacé. Ces circonstances particulières ont entraîné des défis éthiques complexes, qui requièrent une attention immédiate de la part des promoteurs, chercheurs et organismes de réglementation internationaux. Certains promoteurs et organismes de réglementation ont publié des directives sur la manière de gérer les interruptions de recherches cliniques dues à la COVID-19. Elles offrent un bon point de départ pour négocier les écueils au fil de l'évolution de la pandémie, mais elles ne s'appliquent qu'aux chercheurs financés ou administrés par ces instances. Dans le présent document, nous proposons donc des directives destinées à mieux gérer de telles interruptions, au-delà de ce cadre spécifique. Nous soulignons quelques-uns des effets de la pandémie de COVID-19 sur d'autres projets de recherche en cours qui n'ont aucun rapport avec la maladie, et nous donnons des conseils pratiques sur la manière de garantir le bien-être des participants aux études concernées. Enfin, nous parvenons à la conclusion que les législateurs, promoteurs, chercheurs et organismes de réglementation doivent adopter une approche plus souple, afin d'assurer la sécurité des participants tout en préservant l'intégrité des données et en respectant les bonnes pratiques cliniques.

## Resumen

Mientras que los gobiernos se han centrado en la perturbación sin precedentes de la economía global que la enfermedad del coronavirus 2019 (COVID-19) ha causado y en la urgente necesidad de investigar sobre esta enfermedad, otras investigaciones sanitarias se han convertido en víctimas de la pandemia. Las principales actividades de investigación que no están relacionadas con la COVID-19 se han reducido de manera significativa o se han suspendido por completo debido a las restricciones legales relacionadas con la COVID-19 o a los problemas logísticos, de personal u operativos. En la actualidad, el confinamiento o los toques de queda afectan a miles de millones de personas en todo el mundo. Muchos estudios se encuentran ahora en el limbo y el bienestar de decenas de miles de sus participantes está en peligro debido a que se desconoce el calendario de esas medidas restrictivas y está sujeto a cambios. Estas circunstancias han planteado desafíos éticos complejos que merecen una atención urgente por parte de los patrocinadores, los investigadores y los organismos reguladores internacionales. Algunos patrocinadores y organismos reguladores han publicado pautas sobre cómo se deben gestionar las perturbaciones relacionadas con la COVID-19 en la investigación clínica. Aunque estas pautas constituyen un buen punto de partida para hacer frente a los desafíos de la pandemia que está evolucionando, solo se aplican a los investigadores que son financiados o gestionados por estos organismos. En este documento, se ofrecen pautas para gestionar esas perturbaciones que se extienden más allá de estos entornos específicos. Se resaltan algunos de los efectos de la pandemia de la COVID-19 en otros proyectos de investigación en curso que no están relacionados con esta enfermedad y se proporcionan pautas prácticas sobre cómo se debe gestionar el bienestar de los participantes afectados en el estudio. Se concluye que los responsables de formular las políticas, los patrocinadores, los investigadores y los organismos reguladores deben adoptar un enfoque más flexible para garantizar la seguridad de los participantes, al tiempo que se mantiene la integridad de los datos y se cumplen las buenas prácticas clínicas.

## ملخص

بينما كانت الحكومات تركز على الاضطراب غير المسبوق في الاقتصاد العالمي والذي نتج عن مرض فيروس كورونا 2019 (كوفيد-19)، والحاجة الملحة للأبحاث حول كوفيد-19، تأثرت الأبحاث الصحية الأخرى نتيجة للوباء. كما تقلصت بشكل كبير عمليات البحث الرئيسية غير المتعلقة بكوفيد-19، أو توقفت بشكل تام نظراً للقيود القانونية، أو المخاوف اللوجستية أو التشغيلية، أو تلك الخاصة بفرق العمل، والمتعلقة بكوفيد-19. يقع مليارات الأشخاص حول العالم حالياً تحت تأثير عمليات الإغلاق أو حظر التجول. نظراً لأن النطاق الزمني لمثل هذه التدابير التقييدية غير معروف، كما أنه عُرضة للتغيير، فإن العديد من الدراسات أصبحت الآن في غياهب النسيان، كما أن عشرات الآلاف من المشاركين في الدراسة معرضين للخطر. طرحت هذه الظروف تحديات أخلاقية معقدة تستحق الاهتمام العاجل من الرعاة والباحثين والمراقبين الدوليين. قامت جهات راعية وجهات رقابية معينة بنشر توجيهات حول كيفية إدارة الاضطرابات في الأبحاث السريرية، والمتعلقة بكوفيد-19. على الرغم من أن هذه التوجيهات توفر نقطة انطلاق جيدة لاستعراض تحديات الوباء المتزايدة، إلا أنها تنطبق فقط على الباحثين الممولين من جانب هذه الهيئات، أو الخاضعين لإدارتها. نحن نقدم هنا توجيهات حول إدارة مثل هذه الاضطرابات التي تسري خارج إطار هذه الظروف المحددة. نحن نسلط الضوء على بعض آثار وباء كوفيد-19 على المشاريع البحثية الجارية الأخرى، والتي لا علاقة لها بكوفيد-19، كما نقدم إرشادات عملية حول كيفية إدارة رفاهية المشاركين في الدراسة. نحن نستنتج أنه يجب على واضعي السياسات والرعاة والباحثين والمراقبين انتهاج أسلوب أكثر مرونة لضمان سلامة المشاركين، إلى جانب الحفاظ على تكامل البيانات والالتزام بالممارسات السريرية الجيدة.

## 摘要

由于各国政府一直在关注 2019 冠状病毒病（新型冠状病毒肺炎）对全球经济造成的前所未有的破坏，以及对新型冠状病毒肺炎研究的迫切需求，因此导致其他健康研究成为这场大流行下的牺牲品。与新型冠状病毒肺炎无关的重大研究活动要么因为新冠肺炎相关的法律限制，要么出于后勤、人员配备或运营方面的考虑，都被大幅减少或完全暂停。目前全球有数十亿人受到封城或宵禁的影响。由于这种限制性措施的时间范围尚不清楚，而且随时可能会变化，许多研究现在处于停滞状态，数万名研究参与者的福利得不到保障。这些情况引起了复杂的道德挑战，急需国际赞助商、研究人员和监管机构的高度关注。某些赞助商和监管机构已经发布了关于如何管理由新型冠状病毒肺炎导致的临床研究中断事件的指导方针。虽然这些指导方针为应对不断演变的大流行带来的挑战提供了一个很好的起点，但它们只适用于由这些机构资助或管理的研究人员。在本文中，我们提供了管理此类中断事件的指导方针，这些指导方针不止适用于上述特定状况。我们强调了新型冠状病毒肺炎大流行对其他正在进行的与新型冠状病毒肺炎无关的研究项目造成的影响，并就如何管理受影响的研究参与者的福利提供了实用指导。我们的结论是，政策制定者、赞助商、研究人员和监管机构必须采取更灵活的方法，以确保参与者的安全，同时保持数据完整性，并遵守药品临床管理规范。

## Резюме

В то время как внимание правительств стран приковано к беспрецедентной дестабилизации мировой экономики, вызванной коронавирусным заболеванием 2019 года (COVID-19), и к насущной необходимости проведения исследований вируса COVID-19, другие научные исследования в области здравоохранения отошли на второй план из-за пандемии. Крупные научные исследования, не связанные с COVID-19, были значительно сокращены или полностью приостановлены по причине связанных с COVID-19 правовых ограничений или в результате проблем с логистикой, отсутствия специалистов или по соображениям, связанным с текущей деятельностью. Миллиарды людей во всем мире в настоящее время страдают от введенных режимов самоизоляции или комендантского часа. Поскольку сроки таких ограничительных мер неизвестны и могут изменяться, многие исследования в настоящее время находятся в состоянии неопределенности, а благополучие десятков тысяч участников исследований находится под угрозой. Данные обстоятельства привели к возникновению сложных этических проблем, требующих неотложного внимания со стороны международных спонсоров, исследователей и регулирующих государственных органов. Некоторые спонсоры и регулирующие государственные органы опубликовали рекомендации по управлению непредвиденными изменениями в ходе клинических исследований, вызванными COVID-19. Несмотря на то что такие рекомендации являются отличной отправной точкой для решения проблем, связанных с распространением пандемии, они полезны только тем исследователям, которые финансируются или контролируются этими органами. Авторы приводят руководящие принципы по управлению такими непредвиденными изменениями, которые могут применяться к более широкому набору конкретных условий и обстоятельств. Авторы перечисляют некоторые из последствий пандемии COVID-19 для других текущих исследовательских проектов, не связанных с COVID-19, и дают практические рекомендации по управлению благополучием затронутых участников исследований. Авторы приходят к выводу о том, что органы, отвечающие за формирование политики, спонсоры, исследователи и регулирующие государственные органы должны применять более гибкий подход для обеспечения безопасности участников, сохраняя при этом целостность данных и соблюдая передовые клинические практики.

## Introduction

Since the emergence of severe acute respiratory syndrome coronavirus 2 (SARS-CoV-2) in Wuhan, China in November 2019,^1^ and its designation by the World Health Organization (WHO) as a Public Health Emergency of International Concern on 30 January 2020,^2^ the coronavirus disease 2019 (COVID-19) has significantly affected the economic and social fabric of virtually every country. As global cases of COVID-19 rose dramatically, the WHO warned governments in early March 2020 that “allowing uncontrolled spread should not be a choice of any government, as it will harm not only the citizens of that country but affect other countries as well.”^3^ In response to increasing COVID-19 cases, countries and within-country regions imposed restrictions on freedom of movement in an attempt to halt the spread of the disease.

While governments have been focusing on the unprecedented disruption to the global economy^4^ and the urgent need for COVID-19 research,^5^ other health research has become a casualty of the pandemic. Major non-COVID-19 research initiatives around the world have been significantly diminished or suspended entirely because of either COVID-19-related legal restrictions or logistical, staffing or operational concerns.^6^^,^^7^ For example, the global HIV Prevention Trials Network, which is conducting 23 active studies at 65 sites in 14 countries, halted the initiation of new studies, suspended observational studies and postponed all screening and enrolment in ongoing studies.^8^ Similarly, the British National Institute for Health Research suspended all new clinical trials to prioritize COVID-19 studies and enable the redeployment of clinical staff to frontline care.^9^ Since the timescale of restrictive measures, whether full or partial lockdown, is unknown and subject to change, many studies are now in limbo and the welfare of tens of thousands of study participants is at risk. 

These circumstances have introduced complex ethical challenges that merit urgent attention from international sponsors, researchers and regulators. As lockdowns are eased and research activities resume, the primary goal should be the safety and welfare of study participants. Sponsors^10^ and regulatory authorities in some countries^11^^–^^14^ and regions^15^ have published guidance on how the COVID-19-related disruptions of clinical trials should be managed. Although these guidelines are only applicable to investigators funded or governed by these bodies, they do provide a helpful starting point in navigating the challenges of the rapidly evolving pandemic. We provide further guidelines on managing disruptions to health research that will apply beyond the sites and settings covered by the provisional COVID-19 guidance documents published to date.

## Containment measures

Containment measures implemented globally in response to the threat posed by COVID-19 have included issuing advisories on physical distancing and travel, prohibiting social gatherings, imposing travel and visa bans, and discouraging the use of public transport. Some countries or regions have elevated their containment measures with border closures and state-of-emergency decrees, while others have restricted freedom of movement by imposing stay-at-home orders (e.g. multiple states in the United States of America)^16^ or curfews restricting movement to certain hours of the day (e.g. Kenya, Nigeria, Puerto Rico and parts of Thailand).^17^ India, Italy, South Africa and Spain, for example, imposed complete lockdowns lasting several months.^17^ Many countries have transitioned from complete lockdown to risk-adjusted partial lockdown with different degrees of restrictions. 

By their very nature, lockdowns are extremely restrictive with people only being allowed to leave their homes under prescribed circumstances. Full or partial lockdown measures and curfews were affecting more than 4.5 billion people (approximately 58% of the world’s 2020 population of 7.79 billion, estimated by the United Nations)^18^ and almost 2.7 billion workers (representing around 81% of the world’s workforce)^19^ by April 2020. Such disruptions are historically unprecedented in scale, highlighting the equally unparalleled effect on ongoing non-COVID-19 studies. 

## Participant welfare

In most low- and middle-income countries, many participants travel to the site of the study by public transport; however, COVID-19 containment measures that have reduced the availability of such transport (e.g. in India,^20^ Kenya^21^ and South Africa^22^) have made attendance at study sites challenging for participants, delaying passengers and affecting appointment scheduling. Furthermore, because of the confined but generally crowded nature of public transport, by travelling to study sites, study participants expose themselves to the risk of COVID-19 infection. As researchers have an ethical obligation to ensure the safety and welfare of study participants, investigators should explore the feasibility of transporting participants to and from study sites; however, such measures are not without their accompanying budgetary and human resource implications.

Where attendance at the study site is possible, participants face the additional risk of infection from contact with other participants or medical staff; this risk is not likely to be covered by trial insurance. Physical distancing and gathering threshold restrictions must be adhered to, affecting the number of participants able to attend at any time. Even if authorities within a particular country have not made the wearing of face coverings compulsory, study participants and staff should be compelled to wear personal protective equipment when engaged in research-related activities. Such measures have additional cost implications, requiring engagement with study sponsors to secure the required resources. 

Study sites should also develop standard operating procedures for when staff become aware that an offsite study participant (or a member of their household) either has symptoms of COVID-19 or has been in contact with someone with COVID-19 symptoms. Such participants should be instructed not to attend the study site. Investigators should advise participants to inform staff if they test positive for COVID-19 after visiting a study site; other participants and staff who were exposed to that participant may also need to be quarantined and tested.

Since the COVID-19 pandemic will likely disrupt study activities for the foreseeable future, research organizations should utilize existing communication platforms (e.g. text messaging, instant messaging, emails and social media applications) to communicate urgent study-related information regarding, for example, changes to the scheduled site or home visits, provision of study interventions and how trial-related injuries will be managed during public transport restriction measures. Some study participants may not be contactable because of COVID-19 containment measures; for example, participants may be prevented from purchasing top-ups to pay-as-you-go cellular phone plans or prepaid power. In such instances, study staff should persist in trying to contact study participants and must document all attempts to do so. If permitted under local regulations, unscheduled home visits should be considered, but only as a last resort; the potential benefit to the participant of such a visit should be weighed against the potential harm (e.g. privacy implications). Such visits may necessitate a protocol amendment.

## Recruitment and follow-up

Containment strategies have a profound impact on study recruitment, accrual (screening and enrolment) and retention. As well as issues with public transport mentioned above, some participants (or prospective participants) may not be able to attend the study site if a member of their household becomes infected, necessitating the quarantine or self-isolation of the participant.^23^ Studies that involve field work or community surveillance are also affected by the pandemic, as home visits unrelated to COVID-19 surveillance and tracing are typically prohibited during lockdown. 

The retention of study participants is vital to ensure the power and internal validity of longitudinal research, while minimizing loss to follow-up. A study would be negatively affected if participants themselves became infected with COVID-19, or if the area in which the study is located experiences an increasing incidence of COVID-19. In India for example, some health-care facilities advised patients to defer their treatment during lockdown to avoid the risk of COVID-19.^24^ Similarly, hospital admissions in South Africa have fallen since the emergence of COVID-19 because of the decreased utilization of health services as a result of lockdown. Patients in other settings are also avoiding clinical facilities because of fears of nosocomial COVID-19 infection.^25^ In collaboration with the Association of Clinical Research Professionals (which counts more than 13 000 clinical research members across more than 70 countries), a global clinical trial enrolment company conducted a recent survey. Of the clinical trial sites that reported participant dropouts, 80% reported that participants were unwilling to visit the site for study appointments and 52% indicated that participants were wary of interaction with any medical professionals in case they had been in contact with COVID-19 patients.^26^

Participants who acquire COVID-19 and experience long-term effects of the disease, or who defer treatment because of lockdown, may be unable to continue participation in the study, even after restrictions imposed by authorities are eased. Health research studies should therefore consider such factors in their forecasting, and sponsors should be regularly updated on the impact of COVID-19 on study operations. Large numbers of patients who are lost to follow-up could lead to incomplete study outcomes, potentially biasing the study results. Researchers may therefore be required to revaluate their methods of statistical analysis to consider the effect of the pandemic. 

## Study quality

The COVID-19 pandemic may affect data collection and analysis; investigators should therefore outline to regulators and sponsors how they will protect data integrity. Data and safety monitoring boards and committees should closely monitor trial data during the pandemic. For example, studies may have to balance the person-years they already have from existing participants with the potential loss of person-years as a result of disruption by COVID-19. This may necessitate the enrolment of additional participants or the extension of enrolment periods. Such factors could have an impact on budgets, and will require engagement with sponsors and regulators.

In some instances, the effect of the pandemic may be to change the behaviour of participants. In the case of research involving sexual and reproductive health, COVID-19 risk reduction measures will likely affect sexual risk behaviour, such as frequency of sexual encounters, nature of sexual acts, number of partners, concurrent and/or sequential partnerships, and the use of condoms and contraceptives. Such a change in behaviour could affect the treatment and transmission dynamics of specific sexually transmitted diseases and human immunodeficiency virus (HIV) infection, and therefore study outcomes. 

## Intervention studies

The COVID-19-related disruption of intervention studies merits particular attention, especially if the study intervention and/or standard of care comparator is potentially life-saving. Supply of a study intervention may not be sufficient to last until the end of containment measures. If adequate notice is given by authorities of impending containment measures, sufficient supplies of study interventions could be provided to participants; the fact that such measures could be extended without advance notice must also be considered. For example, participants of studies involving sexual and reproductive health are usually provided with condoms and/or oral or injectable contraceptives as a standard of care. Interruptions of such provision could have major implication for study participants, both in terms of risk of pregnancy and disease acquisition. 

Researchers should create contingency plans to continue the provision of such interventions to affected participants. If continued access to such interventions cannot be assured, then under ordinary circumstances regulatory authorities (e.g. the governing research ethics committee) would have to be notified of the consequent protocol violations. Drug regulators, with the input of research ethics committees, data and safety monitoring boards, and study sponsors, should issue guidance on how best to manage such instances.

## Ancillary care

In many studies, investigators provide ancillary care to participants during scheduled study visits. For example, in HIV studies, investigators often treat participants who present with sexually transmitted infections, even if such care is not specified in the study protocol. COVID-19 containment measures will interrupt the provision of such care, affecting the health of the participant. Such an interruption to ancillary care could also have consequences for the health of the public, as the untreated infection of a study participant could be transmitted to others. 

If restrictions imposed by authorities allow for study-related home visits, the pandemic may present ethical challenges related to ancillary care. For instance, if a study participant has symptoms of COVID-19, what ancillary care duties apply? A related dilemma is how to manage instances where a study participant is a pre-symptomatic or asymptomatic carrier of COVID-19, but a member of their household appears to be a symptomatic carrier of the disease. Should ancillary care intended for participants extend to their household members in the context of a public health emergency? Disclosure obligations may also apply in the case of children or elderly people in a study participant’s household. For instance, if test results indicate that a study participant is infected with COVID-19, mandatory disclosure obligations may apply to safeguard the interests of a child or elderly person in their household. 

COVID-19 lockdown and restrictions have also generated food insecurity.^27^^,^^28^ Accordingly, should affected study participants be provided with food as an ancillary care gesture? Domestic violence has increased in some settings during lockdown,^29^ and mental health issues linked to COVID-19 infection or lockdown could persist even after ease of lockdown as a result of financial hardships. What mental health and psychosocial ancillary care^30^^,^^31^ are affected study participants and/or their minor children entitled to? For instance, should study staff refer abused participants to shelters or legal aid? Researchers must devise policies and standard operating procedures to manage such instances.

## Operations and risk mitigation

Risk mitigation must be central to the planning of health research during the COVID-19 pandemic. As well as ensuring the welfare and safety of study participants, research organizations are also responsible for protecting their personnel from exposure to pathogens. Health workers are exposed to many other hazards that increase their risk of COVID-19 infection: long working hours, psychological distress, fatigue, occupational burnout, stigma, and physical and psychological violence.^32^

If researchers are considering offering COVID-19 testing to study participants as part of ancillary care duties, staff must be trained in specimen retrieval and storage, and be provided with appropriate personal protective equipment to perform such duties; sponsors and health authorities should be approached to ensure the provision of required resources. If participants are permitted to visit study sites, designated rooms should be set aside to isolate symptomatic participants as soon as possible after they present at the site. Standard operating procedures should be devised to manage such participants. 

Controlling exposures to occupationally acquired COVID-19 is crucial to the safety of staff. In many settings, health workers are not compelled to put their lives at risk in performing their duties; study staff are therefore justified in refusing to engage in research activities unless provided with relevant personal protective equipment. 

COVID-19 containment measures have significantly affected, and completely paralysed in some cases, local and global supply chains and transport networks. Disruptions to supply chains mean that crucial trial-related supplies, such as study drugs, contraceptives, diagnostic kits and personal protective equipment,^33^ may be in short supply. Researchers and sponsors should put in place measures to manage such instances.

Organizations must also devise contingency plans to cope with the real possibility of key study and support personnel (e.g. clinicians, pharmacists, laboratory workers, drivers, community liaison officers and those tasked with study participant communication) contracting COVID-19. Some organizations may find it challenging to keep fixed-term research study staff employed if it is not possible to conduct the research because of pandemic-related disruptions or restrictions. Such an outcome could have major implications for affected study personnel, especially those at critical points in their careers. Research sponsors must consider providing affected studies with supplemental funding to enable continuation or completion.

Trial insurance will also require consideration; regulatory authorities in many countries require study sponsors and investigators to purchase clinical trial insurance as a precondition to study approval. Such policies are usually set up before trial commencement, and specify the period of insurance cover at the outset. Given trial delays associated with the COVID-19 pandemic, organizations should engage with their broker, managing insurance agent or underwriter to amend policy expiry dates before they lapse.

## Research ethics

Lockdown measures are likely to affect the operations of research ethics committees. In Kenya for example, protocol reviews were suspended until ethics committees could determine how to conduct their work securely, remotely and virtually. Such disruptions to ethics committee operations could cause unpredictable delays to pending or suspended studies. Ethics committees are also likely to be inundated with time-sensitive COVID-19-related protocols. Investigators may engage in such activities in good faith as a response to the pandemic, or they may feel pressured to ensure that their research is regarded as relevant. Either case could inadvertently result in pre-existing research activities being neglected, possibly yielding research that is characterized by redundancy or diminished scientific rigour. As a result of the potential negative implications of such outcomes for enrolled and prospective study participants, ethics committees and regulators must be sensitive to these factors when reviewing research applications.

## Science solidarity

While research ethics imposes obligations on investigators to ensure the welfare of study participants, scientists also have an ethical responsibility to contribute to their country’s response to the pandemic, even if they are not directly involved in COVID-19 research. Non-COVID-19 researchers could, for example, utilize their established community engagement and communication networks to reinforce COVID-19 public health messaging related to physical distancing, hygiene, and containment measures such as quarantine, self-isolation, tracing and surveillance. Research centres that have the capacity to support their country’s response to the pandemic (e.g. by augmenting existing testing or surveillance efforts) should offer to do so. Finally, although such gestures represent solidarity in science and are in the interests of public health and staff and participant safety, they should not be provided at the cost of pre-existing research and scientific rigour.

## Conclusions

The rapidly changing nature and enormity of the COVID-19 crisis represents an unprecedented global challenge to freedom of movement, congregation and enterprise. Cumulatively, these factors represent an unparalleled threat to the conduct of non-COVID-19 health research, a threat that will likely prevail until an efficacious vaccine or cure is developed. Policy-makers, sponsors, researchers and regulators must adopt a more flexible approach to ensure participant safety, while maintaining data integrity and complying with good clinical practices. Failure to embrace a more accommodating attitude in ongoing or prospective studies could irreparably compromise research already affected by the COVID-19 pandemic, and jeopardize the welfare of study participants. Research ethics dictates that we must do everything possible to avoid such an outcome.
